# Long-Term Oncologic Outcomes, Opioid Use, and Complications after Esophageal Cancer Surgery

**DOI:** 10.3390/jcm7020033

**Published:** 2018-02-19

**Authors:** Tak Kyu Oh, Kwhanmien Kim, Sang Hoon Jheon, Sang-Hwan Do, Jung-Won Hwang, Young-Tae Jeon, Kooknam Kim, In-Ae Song

**Affiliations:** 1 Department of Anesthesiology and Pain Medicine, Seoul National University Bundang Hospital, Seongnam 13620, Korea; airohtak@hotmail.com (T.K.O.); 00782@snubh.org (S.-H.D.); jungwon@snubh.org (J.-W.H.); ytjeon@snubh.org (Y.-T.J.); 54271@snubh.org (K.K.); 2 Department of Thoracic and Cardiovascular Surgery, Seoul National University Bundang Hospital, Seongnam 13620, Korea; kimkim0070@snubh.org (K.K.); jheon@snubh.org (S.H.J.)

**Keywords:** esophageal neoplasms, esophagus, anesthesia, analgesia

## Abstract

Effective and adequate opioid use and prevention of postoperative complications are important for enhanced recovery after surgery. We examined the effects of postoperative opioid use and postoperative complications on overall survival and recurrence-free survival after esophageal cancer surgery. This retrospective cohort study analyzed the records of patients diagnosed with esophageal cancer who underwent the Ivor Lewis operation between January 2005 and December 2011. We collected data on total opioid use for 8 days postoperatively, as well as information on postoperative complications (Clavien-Dindo classification). One hundred and twenty-one patients were included in the final analysis. Total opioid use was not significantly associated with overall survival (*p* = 0.520) and recurrence-free survival (*p* = 0.818). In contrast, the hazard ratio of postoperative overall survival was significantly higher with respect to Clavien-Dindo classification 1–2 (hazard ratio: 2.009, *p* = 0.046), 3a–3b (hazard ratio: 5.759, *p* < 0.001), and 4a–5 (hazard ratio: 3.982, *p* = 0.020) complications compared to no complications. Additionally, the hazard ratio of the recurrence-free survival was significantly higher in class 1–2 complications (hazard ratio: 2.336, *p* = 0.028) compared to none. Our study demonstrates that postoperative opioid use is not associated with survival and recurrence-free survival after esophageal cancer surgery, while postoperative complications may increase the hazard ratio for survival and recurrence-free survival.

## 1. Introduction

Although esophageal cancer is a highly fatal cancer, with the sixth highest cancer-related mortality rate [1] research data on esophageal cancer is limited compared to that of other cancers [2]. In general, curative resection is recommended for both limited (T1-2, N0-1, M0) and advanced (T3-4, N0-1, M0) esophageal cancer [3]. However, the recurrence rate remains high even after a successful curative resection, and long-term survival rate remains below 25% [4].

Enhanced recovery after surgery (ERAS), an important care pathway to effectively facilitate early recovery in postoperative patients, has short-term benefits including reduced hospital stay after esophageal cancer surgery [5]. Two key components of ERAS are the maintenance of effective pain control while reducing excessive opioid use and a reduction of postoperative complications. Opioids are reported to increase cancer recurrence after surgery by inducing immunosuppression through their effects on natural killer cell activity [6]. Postoperative complications are also reported to be associated with overall survival (OS) and recurrence rates [7,8]. Therefore, beyond the short-term benefits of ERAS, these two factors are also important with regard to long-term outcomes, particularly oncologic outcomes, which have recently emerged as a topic of emphasis [9].

Postoperative opioid use and postoperative complications after esophageal cancer are closely related because opioid use itself may cause postoperative complications, and patients with postoperative complications generally require more opioids. Thus, it is important to analyze these two factors together with regard to their relationship with OS and recurrence-free survival (RFS) in esophageal cancer patients postoperatively. Accordingly, this study aimed to comprehensively examine the effects of opioid use and postoperative complications on OS and RFS after curative resection in patients with esophageal cancer.

## 2. Materials and Methods

This retrospective cohort study was approved by the Institutional Review Board of the Seoul National University Bundang Hospital (SNUBH) (L2017-246-4). Adults aged ≥19 years old who were diagnosed with esophageal cancer and underwent elective surgical resection (Ivor Lewis operation) at the SNUBH between January 1, 2005 and December 31, 2011 were enrolled in this study. The exclusion criteria were as follows: (1) incomplete medical records; (2) incomplete resection; (3) history of chronic opioid use prior to surgery; and (4) pathologic stages T4, N2, N3, and M1 because a large tumor burden may override the putative effect of opioid and postoperative complications.

The following data were collected from the patients: sex, age, weight (kg), height (cm), postoperative complications (excluding postoperative hoarseness), Clavien-Dindo classification of postoperative complications, location and histologic type of tumor, tumor and lymph node status, adjuvant chemotherapy history, adjuvant radiotherapy history, American Society of Anesthesiologists (ASA) classification, operation time (min), length of hospital stay (days), intensive care unit readmission, epidural analgesia, date of death, date of recurrence, intraoperative remifentanil dose (mcg), and opioid use in the first 8 postoperative days (PODs) (POD 0–7). Postoperative complications were classified according to severity via the Clavien-Dindo classification (7 grades: 1, 2, 3a, 3b, 4a, 4b, and 5) [10]. In consideration of the fact that patients remained hospitalized for at least 9 days after the Ivor Lewis operation, opioid use during the period of 8 days (POD 0–7) was set as the total opioid use. To calculate the opioid use, the standard opioid conversion ratio was used to calculate the morphine equivalent daily dose (MEDD; mg) (Appendix A) [11,12]. Although remifentanil, which was used during and after surgery, is an opioid, its use was collected as a separate independent variable due to the lack of an accurate MEDD conversion ratio. Opioids injected via an epidural route were excluded from the data due to the absence of an accurate MEDD conversion ratio.

All surgical procedures were performed by a highly experienced thoracic surgery team at SNUBH using a standardized technique. Anesthesia was performed by the thoracic anesthesiologist team at SNUBH with a standardized general anesthesia technique using sevoflurane or desflurane and a continuous remifentanil infusion.

Postoperative pain control was generally obtained via fentanyl-based intravenous patient controlled analgesia (PCA) until POD 3, after which oral and intravenous opioids were used based upon the physician’s discretion and patient’s request. Data regarding adjuvant analgesia was not collected for this study because non-steroidal anti-inflammatory drugs are used in several forms, and a conversion formula is lacking. Data regarding acetaminophen use was not collected because it is not generally used until POD 7. All electronic medical record collection was performed by a blinded medical record technician from the medical informatics team at SNUBH. Additionally, the exact date of death of all participants was collected after submitting the rationale and aim of the study and obtaining approval from the Ministry of Public Administration and Security.

The primary aim of this study was to examine how opioid use (POD 0–7) and Clavien-Dindo classification are associated with OS. The secondary aim was to examine how opioid use (POD 0–7) and Clavien-Dindo classification are associated with RFS. For OS, all deaths were censored as an event from the day of surgery, and for RFS, all recurrence dates and deaths were censored as an event from the day of surgery.

### Statistical Methods

Statistical calculations were performed using SPSS (version 12.0, Statistical Package for the Social Sciences, Chicago, IL, USA) and software R (version 3.3.2, A language and environment for statistical computing. R Foundation for Statistical Computing, Vienna, Austria).

Cox regression analysis was used to verify the effects of different covariates and factors on the occurrence of esophageal cancer recurrence. Factor comparison was confirmed by the log-rank test and continuous variables by Cox regression analysis. Variables achieving a probability value of <0.1 in the univariate analysis were subsequently introduced in a multivariate Cox proportional-hazards regression model to identify those variables significantly associated with the occurrence and timing of recurrence. A separate analysis on the subset of patients who were similarly staged as advanced carcinoma was performed to neutralize a beneficial effect on recurrence in early cancer. *p* values <0.05 were considered statistically significant.

## 3. Results

**Figure 1:**
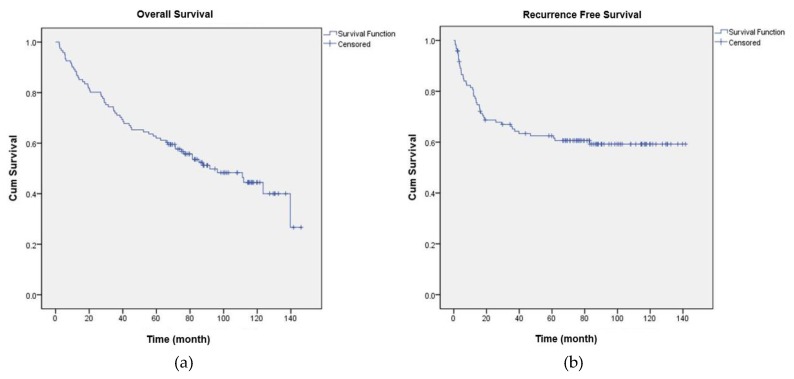
(**a**) Overall survival of all patients, mean (months): 7.2, SD 0.4, 95% confidence interval 6.4–8.0, median (months): 7.5, SD 1.2, 95% confidence interval 5.1–10.0; (**b**) Mean recurrence-free survival of all patients (months): 7.5, SD 0.5, 95% confidence interval 6.6–8.4.

A total of 141 patients were diagnosed with esophageal cancer and underwent the Ivor Lewis operation at SNUBH between January 1, 2005 and December 31, 2011. Eleven patients were excluded based on postoperative pathologic staging (N2, 7 patients; N3, 1 patient; M1, 3 patients), one patient for a history of chronic opioid use, six patients for incomplete resection, and two patients for incomplete medical records regarding opioid use, resulting in a total of 121 patients in the final analysis. Table 1 shows the baseline characteristics of the 121 patients, and Appendix B shows the type and incidence of postoperative complications. The OS and RFS of all patients are shown in Figure 1a,b.

### 3.1. Overall Survival

**Figure 2:**
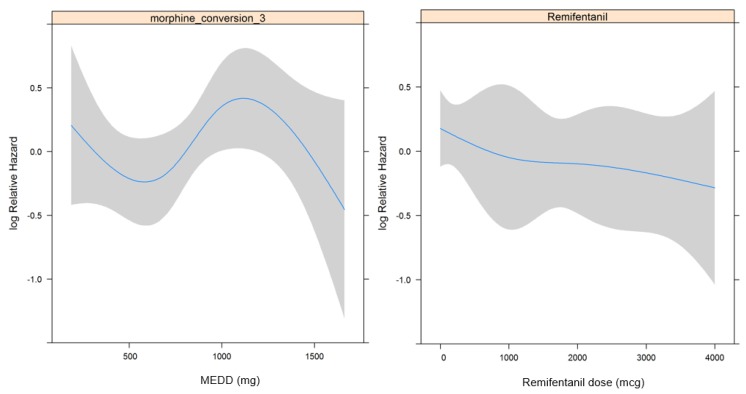
Restricted cubic spline in relation to opioid dosage (**a**) and remifentanil dosage (**b**) with overall survival.

Table 2 shows the results of the univariate and multivariate regression analyses that revealed an association between each factor and OS. In essence, there were no significant associations between OS and intraoperative remifentanil use and opioid use (*p* = 0.235 and 0.520, respectively). Additionally, there was no significant restricted cubic spline (RCS) in relation to remifentanil dosage and opioid dosage (Figure 2a,b). When opioid dosage was divided into quartiles based on the RCS in Figure 2a to analyze its relationship to OS, there was still no statistically significant Hazard Ratio (HR) (*p* = 0.697).

In contrast, patients with Clavien-Dindo classification 1–2, 3a–3b, and 4a–5 complications all had a significantly higher HR compared to those with no complications (None vs. 1–2: HR, 2.009, 95% confidence interval (CI) 1.014–3.979, *p* = 0.046; None vs. 3a–3b: HR, 5.759, 95% CI 2.642–12.554, *p* < 0.001; None vs. 4a–5: HR 3.982, 95% CI 1.244–12.749, *p* = 0.020). Other factors that were associated with OS were age (HR: 1.040, 95% CI 1.005–1.075, *p* = 0.023), body mass index (BMI) (HR: 0.864, 95% CI 0.793–0.941, *p* = 0.001), tumor stage (1 (Ref) vs. 3, HR: 2.737, 95% CI 1.473–5.085, *p* = 0.001), and adjuvant radiotherapy (no (Ref) vs. yes, HR: 2.441, 95% CI 1.310–4.551, *p* = 0.005).

### 3.2. Recurrence Free Survival

**Figure 3:**
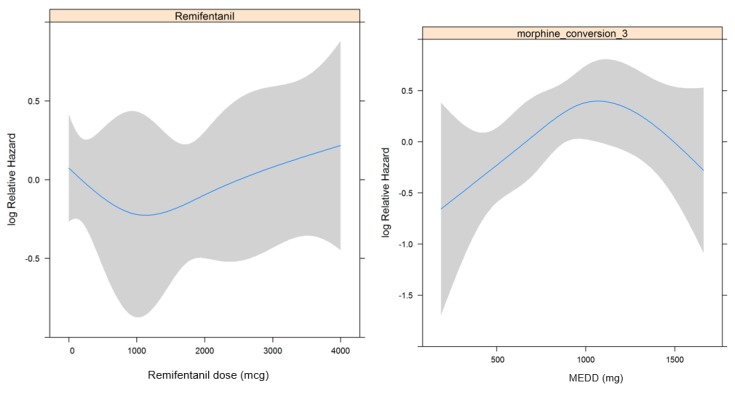
Restricted cubic spline in relation to opioid dosage (**a**) and remifentanil dosage (**b**) with recurrence-free survival.

Table 3 shows the association between each factor and RFS using univariate and multivariate regression analyses. In essence, there were no significant associations between RFS and intraoperative remifentanil use and opioid use (*p* = 0.838 and 0.818, respectively). In addition, the RCS was not significantly associated with remifentanil dosage or opioid dosage (Figure 3a,b). The HR, which was analyzed for RFS by dividing opioid dosage in tertiles based on the RCS shown in Figure 3a (reference: <33%), was significant in the 33–67% tertile (HR 2.385, *p* = 0.024) but was not significant in the >67% tertile (*p* = 0.194).

In terms of the Clavien-Dindo classification, the HR (2.336) (95% CI 1.096–3.984, *p* = 0.028) of RFS was significantly higher in patients with class 1–2 complications compared to those patients without complications, but the HR of patients with class 3a–5 complications was not significantly different from those without complications (*p* = 0.052). Other factors that were significantly associated with RFS were BMI (HR: 0.862, 95% CI 0.789–0.941, *p* = 0.001), tumor stage (1 (Ref) vs. 3, HR: 3.234, 95% CI 1.505–6.947, *p* = 0.003, and adjuvant radiotherapy (no (Ref) vs. yes, HR: 2.768, 95% CI 1.369–5.596, *p* = 0.005).

## 4. Discussion

Our study demonstrates that total opioid use from POD 0–7 and the additional intraoperative use of remifentanil did not affect the postoperative OS and RFS of patients with esophageal cancer. Additionally, we found that postoperative complications, scored using the Clavien-Dindo classification, were intimately associated with the OS (HR 2.009–5.759) and RFS (None vs. grade 1–2 complications, HR: 2.336, *p* = 0.028). These findings are in contrast with those of previous studies that suggested an association between opioid use and cancer recurrence [13,14]. In contrast, our findings are in agreement with previous reports stating that postoperative complications have an impact on OS and RFS after surgery for esophageal cancer [7,8]. The present study is particularly meaningful in that none of our patients was lost to follow-up, and patient death and tumor recurrence were tracked for a minimum of five years. Furthermore, ours is the first study to include remifentanil use as a separate variable in the analysis.

The postoperative period is known to be an important period for recurrence and metastasis after curative surgery [15], and postoperative opioid use, which has protumoral and immunosuppressive effects, is an important issue in oncology [6,13]. Notably, the Ivor Lewis operation for esophageal cancer may require greater postoperative use of opioids because both thoracotomy and laparotomy are performed. Moreover, recent studies have suggested that many esophageal squamous cell carcinoma cells express mu-opioid receptors, which may promote tumor cell growth and facilitate metastasis [16] This has raised concerns that opioid use in the postoperative period after esophageal cancer surgery may have adverse effects on long-term oncologic outcomes, such as OS or RFS. However, in our study, Cox hazard regression analysis revealed that opioid use was not significantly associated with OS and RFS in esophageal cancer patients.

In contrast, postoperative complications were found to be significantly associated with OS and RFS. This association is an important issue with regard to esophageal cancer surgery. Recent studies have reported that the Clavien-Dindo classification provides an effective assessment of complications after esophageal cancer surgery [17]. Postoperative complications using this classification were found to have an impact on patient OS or RFS in two previous studies [7,8], and our findings support the findings of these studies. Particularly, the indirect relationship between postoperative complications and RFS may be a more important issue than the direct relationship between postoperative complications and OS. The reasons for the relationship between RFS and postoperative complications may be multifactorial. First, postoperative complications may induce immunosuppressive effects via the stress response to more severe postoperative pain [18] and through immunosuppression caused by increased opioid use [6]. Further, immune dysfunction caused by malnutrition as a result of various postoperative complications [19] may also be a contributing factor. However, additional studies are needed to substantiate the direct relationship between RFS and postoperative complications.

Factors other than opioid use and postoperative complications were found to be related to OS and RFS in our study. First, BMI had a significant HR for OS (0.864) and RFS (0.862), suggesting that underweight status or malnutrition may have an adverse effect on OS and RFS. BMI was measured immediately before surgery, and our findings show that it is desirable to avoid an underweight status prior to surgery. Second, it is interesting that lymph node status did not have a significant impact on OS and RFS, unlike that of tumor stage. Although patients with 2+ and 3+ lymph node status were excluded from the study because of a large tumor burden, our findings are unusual considering that lymph node status has been associated with recurrence after esophageal cancer surgery [20]. Furthermore, we can explain the high HR of adjuvant radiotherapy for OS and RFS (2.441 and 2.768, respectively) based on the clinical features of patients who received radiotherapy. In general, adjuvant radiotherapy is performed for patients with a more advanced tumor stage or patients whose resection margin is narrower, even if complete resection was performed [21]. Therefore, it is difficult to single out radiotherapy as an independent factor contributing to a shorter OS and RFS. These findings conflict with those of previous studies and should be interpreted carefully. Additional studies are essential.

Our study has a few limitations. First, because this was a retrospective study conducted in a single center, there is a possibility of bias. Second, there may have been differences in surgical and anesthetic techniques in terms of patient management over the period from 2005 through 2011. Third, we used an opioid conversion ratio, but the accuracy of this conversion ratio remains controversial [22]. Finally, we could not examine the association between specific Clavien-Dindo classification grades of complications with the OS or RFS due to the relatively small sample size. Nevertheless, this study is meaningful in that it is the first study to analyze the effects of opioid use (including remifentanil) and postoperative complications as determined by the Clavien-Dindo classification on OS and RFS after esophageal cancer surgery.

In conclusion, our findings showed that postoperative opioid use was not associated with OS and RFS after esophageal cancer surgery, but postoperative complications increased the hazard ratios for OS and RFS. Well-designed prospective studies are needed to substantiate these relationships.

## Figures and Tables

**Table 1:** Distribution for patient characteristics.

Characteristics		Number (%)	Mean (SD)
Total			121
Age (years)			63.5 (8.9)
Body mass index (kg m^−2^)			22.5 (3.2)
Sex: Male		116 (95.9%)	
ASA classification			
	1	28 (23.1%)	
	2	77 (63.6%)	
	3	16 (13.2%)	
Operation time (min)			416.7 (127.1)
Estimated blood loss (mL)			512.4 (573.8)
Location of tumor			
	Upper	24 (19.8%)	
	Middle	18 (14.9%)	
	Lower	79 (65.3%)	
Pathology of tumor			
	Squamous cell carcinoma	111 (91.7%)	
	Adenocarcinoma	10 (8.3%)	
Pathologic cancer stage			
	IA, IB	40 (33.0%)	
	IIA, IIB	58 (47.0%)	
	IIIA, IIIB, IIIC	23 (19.0%)	
Pathologic tumor stage			
	T1	50 (41.3%)	
	T2	25 (20.7%)	
	T3	46 (38.0%)	
Pathologic lymph node stage			
	N0	72 (59.5%)	
	N1	49 (40.5%)	
Adjuvant chemotherapy		43 (35.5%)	
Adjuvant radiotherapy		19 (15.7%)	
Postoperative complication		46 (38.0%)	
Clavien-dindo classification			
	None	75 (61.2%)	
	1 + 2	18 (14.9%)	
	3a + 3b	18 (14.9%)	
	4a + 4b + 5	10 (8.3%)	
ICU readmission		10 (8.3%)	
Epidural analgesia		7 (5.8%)	
Intraoperative remifentanil dose (mcg)			1284.6 (1363.8)
Opioid consumption in POD 0–7, MEDD (mg)			884.0 (458.5)

SD, Standard Deviation; ASA, American Society of Anesthesiologists; ICU, Intensive Care Unit; POD, Postoperative Day; MEDD, Morphine Equivalent Daily.

**Table 2:** Univariate and multivariate cox regression analysis for overall survival in esophageal cancer patients.

		Univariate Cox Regression Model		Multivariate Cox Regression Model	
Variables		Hazard ratio (95% CI)	*p value*	Hazard Ratio (95% CI)	*p value*
Age		1.028 (0.998–1.060)	0.072	1.040 (1.005–1.075)	0.023
Sex: male		1.247 (0.304–5.108)	0.759		
Body mass index		0.879 (0.811–0.954)	0.002	0.864 (0.793–0.941)	0.001
ASA classification					
	2 (vs 1)	2.197 (1.030–4.684)	0.042	2.259 (0.984–5.186)	0.055
	3 (vs 1)	2.919 (1.191–7.151)	0.019	1.938 (0.752–4.991)	0.171
Epidural analgesia		1.756 (0.702–4.392)	0.229		
Length of hospital stay		1.008 (0.999–1.017)	0.074	0.996 (0.982–1.010)	0.562
ICU readmission		1.362 (0.585–3.168)	0.473		
Operation time		1.001 (1.000–1.003)	0.136		
Estimated blood loss		1.000 (1.000–1.000)	0.783		
Pathology of tumor: squamous cell carcimoma		1.247 (0.452–3.439)	0.670		
Location of tumor					
	Middle (vs upper)	1.371 (0.645–2.912)	0.412		
	Lower (vs upper)	0.622 (0.344–1.125)	0.116		
Pathologic cancer stage					
	IIA, IIB (vs IA, IB)	2.514 (1.359–4.651)	0.003	1.841 (0.889–3.815)	0.101
	IIIA, IIIB, IIIC (vs IA, IB)	2.271 (1.076–4.793)	0.031	1.920 (0.877–4.204)	0.103
Pathologic tumor stage					
	T2 (vs T1)	1.288 (0.621–2.761)	0.496	1.570 (0.716–3.446)	0.260
	T3 (vs T1)	2.474 (1.418–4.316)	0.001	2.737 (1.473–5.085)	0.001
Pathologic lymph node stage					
	N1 (vs N0)	1.315 (0.799–2.165)	0.281		
Adjuvant chemotherapy		1.452 (0.878–2.402)	0.146		
Adjuvant radiotherapy		3.035 (1.723–5.345)	<0.001	2.441 (1.310–4.551)	0.005
Intraoperative remifentanil (mcg)		1.000 (1.000–1.000)	0.235		
Opioid consumption in POD 0–7, MEDD (mg)		1.000 (0.999–1.001)	0.520		
	≤630 (25%)	1	0.697		
	≤843 (50%)	0.923 (0.452–1.884)			
	≤1125 (75%)	1.333 (0.681–2.612)			
	> 1125	0.950 (0.456–1.982)			
Clavien-dindo classification					
	1 + 2 (vs no complication)	2.182 (1.123–4.239)	0.021	2.009 (1.014–3.979)	0.046
	3a + 3b (vs no complication)	4.351 (2.324–8.145)	<0.001	5.759 (2.642–12.554)	<0.001
	4a + 4b + 5 (vs no complication)	3.137 (1.358–7.247)	0.007	3.982 (1.244–12.749)	0.020

Multivariate cox regression model: Variables with *p*-value <0.1 from the univariable cox regression model were included in the multivariable model CI, confidence interval; ASA, American Society of Anesthesiologists; ICU, intensive care unit; MEDD, morphine equivalent daily dose.

**Table 3:** Univariate and multivariate Cox regression analysis for recurrence-free survival in esophageal cancer patients.

		Univariate Cox Regression Model		Multivaiate Cox Regression Model	
Variables		Hazard Ratio (95% CI)	*p value*	Hazard Ratio (95% CI)	*p value*
Age		1.010 (0.976–1.045)	0.576		
Sex: male		0.914 (0.222–3.770)	0.901		
Body Mass Index		0.843 (0.771–0.923)	<0.001	0.862 (0.789–0.941)	0.001
ASA classification					
	2 (vs 1)	2.473 (1.039–5.885)	0.041	1.783 (0.726–4.381)	0.207
	3 (vs 1)	1.954 (0.629–6.067)	0.246	1.100 (0.342–3.534)	0.873
Epidural analgesia		1.695 (0.608–4.725)	0.313		
Length of hospital stay		1.003 (0.990–1.015)	0.684		
ICU readmission		1.265 (0.393–4.075)	0.693		
Operation time		0.999 (0.997–1.002)	0.552		
Estimated blood loss		1.000 (0.999–1.000)	0.770		
Pathology of tumor : Squamous cell carcimoma		1.362 (0.423–4.387)	0.605		
Location of tumor					
	Middle (vs upper)	1.305 (0.540–3.151)	0.555		
	Lower (vs upper)	0.691 (0.343–1.394)	0.302		
Pathologic cancer stage					
	IIA, IIB (vs IA, IB)	2.574 (1.199–5.528)	0.015	2.094 (0.807–5.435)	0.129
	IIIA, IIIB, IIIC (vs IA, IB)	3.748 (1.595–8.803)	0.002	1.885 (0.673–5.283)	0.228
Pathologic tumor stage					
	T2 (vs T1)	1.563 (0.638–3.827)	0.328	1.489 (0.557–3.984)	0.428
	T3 (vs T1)	4.067 (2.048–8.075)	<0.001	3.234 (1.505–6.947)	0.003
Pathologic lymph node stage					
	N1 (vs N0)	1.486 (0.838–2.636)	0.175		
Adjuvant chemotherapy		2.657 (1.491–4.734)	<0.001	1.447 (0.730–2.870)	0.290
Adjuvant radiotherapy		3.859 (2.062–7.220)	<0.001	2.768 (1.369–5.596)	0.005
Intraoperative remifentanil (mcg)		1.000 (1.000–1.000)	0.838		
Opioid consumption in POD 0–7, MEDD (mg)			0.818		
	≤684 (33%)	1			
	≤1020.6 (67%)	2.385 (1.122–5.072)	0.024		
	>1020.6	1.689 (0.766–3.722)	0.194		
Clavien-dindo classification					
	1 + 2 (vs no complication)	2.622 (1.269–5.417)	0.009	2.336 (1.096–3.984)	0.028
	3a + 3b + 4a + 4b + 5 (vs no complication)	2.457 (1.255–4.812)	0.009	2.040 (0.994–4.183)	0.052

Multivariate cox regression model: Variables with *p*-value < 0.1 from the univariable cox regression model were included in the multivariable model. CI, confidence interval; ASA, American Society of Anesthesiologists; ICU, intensive care unit; MEDD, morphine equivalent daily dose.

## Appendix A

**Table A1:** Equianalgesic opioid conversion table.

Opioid	Administration Route	Dose Equivalent to 10 mg of Oral Morphine (mg)
Morphine	Oral	10
Morphine	Intravenous	3.3
Morphine	Epidural	0.33
Hydromorphone	Oral	2
Fentanyl	Intravenous	0.03
Oxycodone	Oral	7
Codeine	Oral	80
Tramadol	Oral	40

## Appendix B

**Table A2:** Postoperative complications after esophageal cancer surgery.

Postoperative Complication	n	%
Pulmonary complication	28	38%
Anastomtic leakage	13	18%
Wound infection of dehiscence	18	25%
Urinary tract infection	1	1%
Chylothorax	2	3%
New-onset arrythmia	2	3%
Ileus	2	3%
Acute kidney injury	4	5%
Graft failure	3	4%

## References

[1] Enzinger P.C., Mayer R.J. (2003). Esophageal cancer. N. Engl. J. Med..

[2] Zhang Y. (2013). Epidemiology of esophageal cancer. World J. Gastroenterol..

[3] Worni M., Castleberry A.W., Gloor B., Pietrobon R., Haney J.C., D’Amico T.A., Akushevich I., Berry M.F. (2014). Trends and outcomes in the use of surgery and radiation for the treatment of locally advanced esophageal cancer: A propensity score adjusted analysis of the surveillance, epidemiology, and end results registry from 1998 to 2008. Dis. Esophagus.

[4] Stahl M., Budach W., Meyer H.J., Cervantes A., ESMO Guidelines Working Group (2010). Esophageal cancer: Clinical practice guidelines for diagnosis, treatment and follow-up. Ann. Oncol..

[5] Blom R.L., van Heijl M., Bemelman W.A., Hollmann M.W., Klinkenbijl J.H., Busch O.R., van Berge Henegouwen M.I. (2013). Initial experiences of an enhanced recovery protocol in esophageal surgery. World J. Surg..

[6] Cata J.P., Gottumukkala V., Sessler D.I. (2011). How regional analgesia might reduce postoperative cancer recurrence. Eur. J. Pain Suppl..

[7] Lerut T., Moons J., Coosemans W., Van Raemdonck D., de Leyn P., Decaluwe H., Decker G., Nafteux P. (2009). Postoperative complications after transthoracic esophagectomy for cancer of the esophagus and gastroesophageal junction are correlated with early cancer recurrence: Role of systematic grading of complications using the modified clavien classification. Ann. Surg..

[8] Markar S., Gronnier C., Duhamel A., Mabrut J.Y., Bail J.P., Carrere N., Lefevre J.H., Brigand C., Vaillant J.C., Adham M. (2015). The impact of severe anastomotic leak on long-term survival and cancer recurrence after surgical resection for esophageal malignancy. Ann. Surg..

[9] Bugada D., Bellini V., Fanelli A., Marchesini M., Compagnone C., Baciarello M., Allegri M., Fanelli G. (2016). Future perspectives of eras: A narrative review on the new applications of an established approach. Surg. Res. Pract..

[10] Clavien P.A., Barkun J., de Oliveira M.L., Vauthey J.N., Dindo D., Schulick R.D., de Santibanes E., Pekolj J., Slankamenac K., Bassi C. (2009). The Clavien-Dindo classification of surgical complications: Five-year experience. Ann. Surg..

[11] Benzon H., Rathmell J.P., Wu C.L., Turk D.C., Argoff C.E. (2008). Raj’s Practical Management of Pain.

[12] Jacox A., Carr D., Payne R., Berde C., Breitbart W., Cain J., Chapman C., Cleeland C., Ferrell B., Finley R. (1994). Management of cancer pain-adults. Am. Fam. Physician.

[13] Cata J.P., Keerty V., Keerty D., Feng L., Norman P.H., Gottumukkala V., Mehran J.R., Engle M. (2014). A retrospective analysis of the effect of intraoperative opioid dose on cancer recurrence after non-small cell lung cancer resection. Cancer Med..

[14] Maher D.P., Wong W., White P.F., McKenna R., Rosner H., Shamloo B., Louy C., Wender R., Yumul R., Zhang V. (2014). Association of increased postoperative opioid administration with non-small-cell lung cancer recurrence: A retrospective analysis. Br. J. Anaesth..

[15] Sessler D.I. (2008). Does regional analgesia reduce the risk of cancer recurrence? A hypothesis. Eur. J. Cancer. Prev..

[16] Zhang Y.F., Xu Q.X., Liao L.D., Xu X.E., Wu J.Y., Wu Z.Y., Shen J.H., Li E.M., Xu L.Y. (2015). Association of mu-opioid receptor expression with lymph node metastasis in esophageal squamous cell carcinoma. Dis. Esophagus.

[17] Umezawa H., Nakao J., Matsutani T., Kuwahara H., Taga M., Ogawa R. (2016). Usefulness of the Clavien-Dindo classification in understanding the limitations and indications of larynx-preserving esophageal reconstruction. Plast. Reconstr. Surg. Glob. Open.

[18] Beilin B., Shavit Y., Trabekin E., Mordashev B., Mayburd E., Zeidel A., Bessler H. (2003). The effects of postoperative pain management on immune response to surgery. Anesth. Analg..

[19] Bourke C.D., Berkley J.A., Prendergast A.J. (2016). Immune dysfunction as a cause and consequence of malnutrition. Trends Immunol..

[20] Kunisaki C., Makino H., Kimura J., Oshima T., Fujii S., Takagawa R., Kosaka T., Ono H.A., Akiyama H. (2010). Impact of lymph-node metastasis site in patients with thoracic esophageal cancer. J. Surg. Oncol..

[21] Chen H., Wang Z., Yang Z., Shang B., Liu X., Chen G. (2013). Prospective study of adjuvant radiotherapy on preventing lymph node metastasis after Ivor-Lewis esophagectomy in esophageal cancer. Ann. Surg. Oncol..

[22] O’Bryant C.L., Linnebur S.A., Yamashita T.E., Kutner J.S. (2008). Inconsistencies in opioid equianalgesic ratios: Clinical and research implications. J. Pain. Palliat. Care. Pharmacother..

